# Real-world experience with cabazitaxel in patients with metastatic castration-resistant prostate cancer: a final, pooled analysis of the compassionate use programme and early access programme

**DOI:** 10.18632/oncotarget.27031

**Published:** 2019-06-25

**Authors:** Zafar Malik, Axel Heidenreich, Sergio Bracarda, Alexandros Ardavanis, Philip Parente, Hans-Joerg Scholz, Ayse Ozatilgan, Evelyne Ecstein-Fraisse, Simon Hitier, Giuseppe Di Lorenzo

**Affiliations:** ^1^The Clatterbridge Cancer Centre NHS Foundation Trust, Wirral, UK; ^2^Department of Urology, Uro-Oncology, Robot-Assisted and Specialized Urologic Surgery, University Hospital Cologne, Cologne, Germany; ^3^Azienda USL Toscana Sud-Est, Istituto Toscana Tumori (ITT), Ospedale San Donato, Arezzo, Italy; ^4^Oncology Hospital AGIOS SAVVAS Oncology Clinic, Athens, Greece; ^5^ECRU-Oncology, Victoria, Australia; ^6^Asklepios Klink GmbH Weissenfels, Weissenfels, Germany; ^7^Sanofi, Cambridge, Massachusetts, USA; ^8^Sanofi, Paris, France; ^9^Sanofi, Chilly-Mazarin, France; ^10^Department of Clinical Medicine and Surgery, University of Naples Federico II, Napoli, Italy

**Keywords:** mCRPC, cabazitaxel, CUP, EAP, real-world

## Abstract

**Background:**

Cabazitaxel is a second-generation taxane approved for use in patients with metastatic castration-resistant prostate cancer (mCRPC) previously treated with docetaxel. Early access programmes were established to allow eligible patients with mCRPC access to cabazitaxel before regulatory approval.

**Materials and Methods:**

The primary objective was to allow access to cabazitaxel before commercial availability for patients with mCRPC whose disease had progressed during or after chemotherapy with docetaxel; the secondary objective was overall safety. Patients received cabazitaxel 25 mg/m^2^ on Day 1 of a 21-day cycle, with daily oral 10 mg prednisone/prednisolone. G-CSF was administered per ASCO guidelines.

**Results:**

In total, 1432 patients received cabazitaxel across 41 countries between 2010 and 2014 (median 6.0 treatment cycles [range 1–49]). The most frequently occurring treatment-emergent adverse events (TEAEs) possibly related to treatment were diarrhoea (33.3%), fatigue (25.4%) and anaemia (23.7%); the most frequently occurring possibly related Grade 3/4 TEAEs were neutropenia (18.7%) and febrile neutropenia (6.9%). G-CSF was administered in ≥ 1 cycle in 64% of patients (10.1% therapeutic use; 57.8% prophylactic use; 9.7% both uses).

**Conclusion:**

The safety profile of cabazitaxel in this pooled analysis of two cabazitaxel early access programmes was manageable and consistent with previous Phase III trials (TROPIC, PROSELICA).

## INTRODUCTION

Prostate cancer is the second most commonly occurring cancer in men worldwide [1]. Early diagnosis is typically associated with better prognosis; however, 10–20% of patients progress to a castration-resistant state, after castration-sensitive disease, within 5 years [2], while 5–21% of patients present with distant metastases at diagnosis [3, 4]. Docetaxel was approved as a first-line treatment for metastatic castration-resistant prostate cancer (mCRPC, previously defined as hormone-refractory prostate cancer) in 2004 following completion of two Phase III trials: TAX-327 and SWOG-9916, in which docetaxel was associated with a significant improvement in overall survival compared with mitoxantrone/prednisone [5, 6].

Cabazitaxel is a second-generation taxane agent, specifically designed to overcome resistance to docetaxel. Cabazitaxel was approved in 2010 based on its survival advantage of 2.4 months over mitoxantrone (hazard ratio 0.70; 95% confidence interval: 0.59–0.83; *p* < 0.0001) in the Phase III TROPIC trial [7]. A substantial proportion of patients in TROPIC had shown early progression during previous treatment with docetaxel: 72% of patients had progressed within 3 months of their last dose of docetaxel, and 30% had progressed during treatment itself. In addition, 25% of patients had poor prognosis shown by visceral disease [7]. Prior to the TROPIC study, patients who progressed would have either continued on docetaxel, switched to palliative, non-chemotherapeutic treatments or received no treatment. In a follow-up survival analysis of TROPIC (cut-off 10 March 2010), the probability of survival at ≥ 24 months was 27% for cabazitaxel compared with 16% for mitoxantrone (hazard ratio 0.72; 95% confidence interval: 0.61–0.84; *p* < 0.0001); this analysis also highlighted the benefit of cabazitaxel over mitoxantrone across multiple patient sub-groups [8].

In TROPIC, there was a higher incidence of Grade ≥ 3 treatment-emergent adverse events (TEAEs) in the cabazitaxel group compared with the mitoxantrone group: laboratory-confirmed neutropenia (82% vs. 58%), febrile neutropenia (8% vs. 1%) and diarrhoea (6% vs. <1%) [7]. The multicentre TROPIC study was conducted at 146 centres in 26 countries – each with varied experience of using chemotherapy for the treatment of mCRPC and different proactive and reactive plans for the management of TEAEs [7]. No primary prophylaxis, defined as treatment before or during Cycle 1 with granulocyte colony-stimulating factor (G-CSF), was initially offered for neutropenia in the TROPIC study. However, after the occurrence of neutropenia-related deaths (7 patients [2%] receiving cabazitaxel in TROPIC overall), the Independent Data Monitoring Committee recommended that investigators strictly follow protocol-recommended dose modifications and treatment of neutropenia, as per American Society of Clinical Oncology (ASCO) guidelines, and that prophylactic G-CSF be used at the discretion of the investigator, except during Cycle 1 of the treatment. After this recommendation, no further deaths occurred in TROPIC and the application of ASCO guidelines in all subsequent trials was encouraged. Later studies have since suggested that prophylactic G-CSF could be used to prevent and manage neutropenia [7, 9–14].

Based on the positive results of TROPIC and the unmet medical need at the time, two studies (compassionate use programme [CUP] and early access programme [EAP]) were established to enable eligible patients with mCRPC early access to cabazitaxel (prior to its commercial availability), and to document and verify its overall safety. Health-related quality-of-life (HRQoL) data were also collected in certain countries, such as Canada, Australia and the UK.

## RESULTS

### Patient population

**Figure 1:**
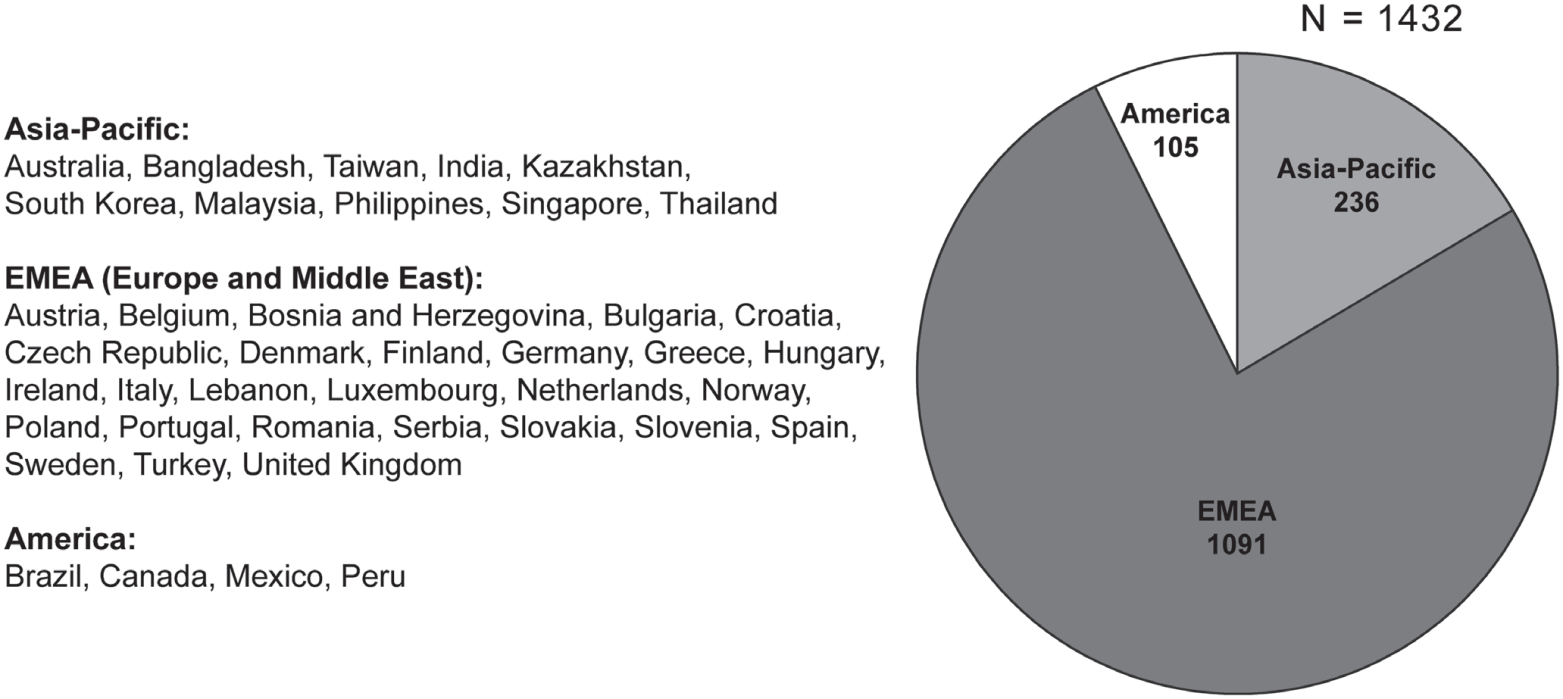
Patient enrolment by region.

A total of 1432 patients were enrolled: in CUP, 451 patients were enrolled from 12 countries between July 2010 and May 2013; in EAP, 981 patients were enrolled from 29 countries between December 2010 and December 2014 (Figure 1). Patients had a median age of 68.0 years (range 42–89; Table 1). Most patients had an Eastern Cooperative Oncology Group performance status (ECOG PS) of 0 or 1 (91.1%). The median time from mCRPC diagnosis to inclusion in CUP or EAP (Q1–Q3) was 21.3 months (13.0–36.4). Baseline disease characteristics were similar across the CUP and EAP programmes.

**Table 1:** Patient baseline characteristics

	CUP (*N* = 451)	EAP (*N* = 981)	CUP/EAP pooled (*N* = 1432)
**Mean age (range), years**	67.4 (43–84)	68.2 (42–89)	68.0 (42–89)
< 65 years, *n* (%)	148 (32.8)	298 (30.4)	446 (31.1)
65–75 years, *n* (%)	222 (49.2)	461 (47.0)	683 (47.7)
≥ 75 years, *n* (%)	81 (18.0)	222 (22.6)	303 (21.2)
**ECOG PS, *n* %**			
0	174 (38.7)	414 (42.2)	588 (41.1)
1	231 (51.3)	485 (49.4)	716 (50.0)
2	45 (10.0)	82 (8.4)	127 (8.9)
**Metastatic sites^a^**			
0	2 (0.4)	0 (0.0)	2 (0.1)
1	179 (39.7)	391 (39.9)	570 (39.8)
≥ 2	270 (59.9)	590 (60.1)	860 (60.1)
**Time since mCRPC diagnosis to inclusion, median (Q1–Q3), months**	18.4 (11.0–31.7)	22.7 (13.8–37.8)	21.3 (13.0–36.4)
**Cycles of last docetaxel use, median (Q1–Q3), *n***	9.0 (6.0–12.0)	8.0 (6.0–10.0)	8.0 (6.0–10.0)
**Time from last docetaxel dose to first cabazitaxel dose, median (Q1–Q3), months**	4.4 (2.2–9.4)	5.5 (2.6–11.6)	5.3 (2.4–10.9)
**Time from last docetaxel dose to first cabazitaxel dose by class, *n* (%)**			
≤ 6 months	269 (60.3)	513 (52.6)	782 (55.0)
> 6 months	177 (39.7)	463 (47.4)	640 (45.0)
**Time from last docetaxel dose to last progression by class, *n* (%)**			
< 0 months (last administration occurred after progression)	108 (24.3)	128 (13.1)	236 (16.6)
< 3 months since last docetaxel dose	165 (37.1)	315 (32.3)	480 (33.8)
3–6 months since last docetaxel dose	62 (13.9)	172 (17.7)	234 (16.5)
≥ 6 months since last docetaxel dose	110 (24.7)	359 (36.9)	469 (33.1)

^a^Metastatic site refers to an organ system (such as bone, lymph node, lungs, etc.) and not a single lesion. ECOG PS, Eastern Cooperative Oncology Group performance status; Q1–Q3, interquartile range.

The median number of cycles of most recent docetaxel administration (Q1–Q3) was 8.0 (6.0–10.0; Table 1) and the median time from last docetaxel dose to first cabazitaxel dose (Q1–Q3) was 5.3 months (2.4–10.9). In this population, a third of patients (33.8%) had progressed within 3 months after last docetaxel dose (16.6% had progressed before last docetaxel dose).

### Treatment exposure

Patients received a median of 6.0 cycles (range 1–49) of cabazitaxel (Table 2). Approximately 1 in 4 patients (*n* = 347; 24.2%) received 10 or more cycles of treatment. The median duration of treatment (Q1–3) was 18.6 weeks (12.0–29.6): the shortest duration was 3.0 weeks (one cycle) and the longest was 153.1 weeks. The median intended cumulative dose (Q1–Q3) was 150 mg/m^2^ (85–225); the median actual cumulative dose (Q1–Q3) was 147.3 mg/m^2^ (84.0–219.4). The median intended dose intensity (Q1–Q3) was 8.1 mg/m^2^/week (7.4–8.3); the median actual dose intensity (Q1–Q3) was 7.8 mg/m^2^/week (7.1–8.3). Treatment discontinuation resulted from disease progression in 641 patients (44.8%), TEAEs in 352 patients (24.6%), the investigator’s decision in 208 patients (14.5%), the commercial availability of cabazitaxel in 83 patients (5.8%) and other reasons in 148 patients (10.3%, including patient’s decision in 70 patients [4.8%]).

**Table 2:** Use of cabazitaxel during the study

	CUP (*N* = 451)	EAP (*N* = 981)	CUP/EAP pooled (*N* = 1432)
**Median number of cycles (Q1–Q3) [range]**	5.0 (3.0–8.0) [1.0–34.0]	6.0 (4.0–10.0) [1.0–49.0]	6.0 (4.0–9.0) [1.0–49.0]
**Patients who received ≥ 10 cycles of treatment, *n* (%) [range]**	82 (18.2)	265 (27.0)	347 (24.2)
**Median intended dose intensity, mg/m²/week (Q1–Q3) [range]**	8.3 (7.6–8.3) [3.7–8.6]	8.0 (7.3–8.3) [4.5–8.9]	8.1 (7.4–8.3) [3.7–8.9]
**Median actual dose intensity, mg/m²/week (Q1–Q3) [range]**	7.9 (7.3–8.3) [3.6–8.9]	7.8 (7.1–8.2) [3.1–9.1]	7.8 (7.1–8.3) [3.1–9.1]
**Median intended cumulative dose, mg/m^2^(Q1–Q3) [range]**	125 (75–200) [20–823]	150 (100–225) [20–1010]	150 (85–225) [20–1010]
**Median actual cumulative dose, mg/m^2^(Q1–Q3) [range]**	124.4 (73.9–197.7) [19.2–817.1]	150.3 (95.9–224.7) [19.1–1031.7]	147.3 (84.0–219.4) [19.1–1031.7]
**Duration of exposure, median (Q1–Q3) [range], weeks**	15.9 (9.1–26.7) [3.0–104.1]	21.0 (12.1–30.0) [3.0–153.1]	18.6 (12.0–29.6) [3.0–153.1]

Q1–Q3, interquartile range.

### G-CSF use for neutropenia

At Cycle 1, 599 patients (41.8%) received prophylactic G-CSF for neutropenia, 95 patients (6.6%) received therapeutic G-CSF and 78 patients (5.4%) received both a prophylactic and therapeutic dose (Table 3). Overall, 827 patients (57.8%) received prophylactic G-CSF for neutropenia at any cycle, and 145 patients (10.1%) received therapeutic G-CSF (139 patients [9.7%] received both a prophylactic and therapeutic dose). The use of prophylactic G-CSF was more common in patients > 65 years of age (Table 3).

**Table 3:** G-CSF use during the study

	CUP *N* = 451	EAP *N* = 981	CUP/EAP pooled *N* = 1432		
Number of patients with G-CSF administration, *n* (%)			All ages	≤ 65 years of age	> 65 years of age
**Total G-CSF at Cycle 1**	214 (47.5)	558 (56.9)	772 (53.9)	233 (44.4)	539 (59.4)
Therapeutic	29 (6.4)	66 (6.7)	95 (6.6)	31 (5.9)	64 (7.1)
Prophylactic	137 (30.4)	462 (47.1)	599 (41.8)	182 (34.7)	417 (46.0)
Therapeutic + prophylactic	48 (10.6)	30 (3.1)	78 (5.4)	20 (3.8)	58 (6.4)
**Total G-CSF in at least one cycle, *n* (%)**	248 (55.0)	669 (68.2)	917 (64.0)	290 (55.2)	627 (69.1)
Therapeutic	40 (8.9)	105 (10.7)	145 (10.1)	50 (9.5)	95 (10.5)
Prophylactic	211 (46.8)	616 (62.8)	827 (57.8)	259 (49.3)	568 (62.6)
Therapeutic + prophylactic	72 (16.0)	67 (6.8)	139 (9.7)	36 (6.9)	103 (11.4)

G-CSF, granulocyte-colony stimulating factor.

### Safety

TEAEs considered possibly related to study treatment were observed more often during the first cycle of cabazitaxel. In all cycles, the most frequently occurring TEAEs possibly related to study treatment (all grades) were diarrhoea (33.3%), fatigue (25.4%), anaemia (23.7%), nausea (22.4%) and neutropenia (22.1%, Table 4). The most frequently occurring Grade 3/4 TEAEs considered possibly related to study treatment were neutropenia (18.7%), febrile neutropenia (6.9%), leukopenia (6.4%), anaemia (4.9%) and fatigue (4.3%). Grade 3/4 diarrhoea occurred in 3.6% of patients and Grade 3/4 nausea in 1.3% (Table 4). Treatment-related neutropenic sepsis occurred in 24 patients (1.7%; Grade 3/4 in 22 patients [1.5%]); sepsis occurred in 13 patients (0.9%; all Grade 3/4) and febrile neutropenia occurred in 101 patients (7.1%; Grade 3/4 in 99 patients [6.9%]). Only 5 cases (0.3%) of treatment-related Grade 3/4 peripheral neuropathy occurred (3 cases of neuropathy and 2 cases of sensory neuropathy); these cases of Grade 3–4 neuropathy may have either occurred *de novo* or as a worsening of docetaxel-induced Grade 1–2 neuropathy. All-cause serious adverse events (SAEs) occurred in 38.6% of patients and Grade 3/4 SAEs occurred in 31.4% (CUP [33.9% and 27.1%]; EAP [40.8% and 33.3%]). The most frequently occurring Grade 3/4 SAEs were febrile neutropenia (5.9%), neutropenia (2.9%), diarrhoea (1.6%), neutropenic sepsis (1.5%) and disease progression (1.5%).

**Table 4:** Treatment-related TEAEs of any grade occurring in > 10% of patients and treatment-related Grade 3/4 TEAEs occurring in > 3% of patients

Patients, *n* (%)	CUP (*N* = 451)	EAP (*N* = 981)	CUP/EAP pooled (*N* = 1432)
**Any treatment-related, any grade TEAE (> 10%)**	329 (72.9)	846 (86.2)	1175 (82.1)
Diarrhoea	117 (25.9)	360 (36.7)	477 (33.3)
Fatigue	93 (20.6)	271 (27.6)	364 (25.4)
Anaemia	99 (22.0)	241 (24.6)	340 (23.7)
Nausea	73 (16.2)	248 (25.3)	321 (22.4)
Neutropenia	92 (20.4)	225 (22.9)	317 (22.1)
Decreased appetite	51 (11.3)	153 (15.6)	204 (14.2)
Vomiting	43 (9.5)	154 (15.7)	197 (13.8)
Asthenia	16 (3.5)	181 (18.5)	197 (13.8)
**Any treatment-related, Grade 3/4 TEAE (> 3%)**	186 (41.2)	432 (44.0)	618 (43.2)
Neutropenia	76 (16.9)	192 (19.6)	268 (18.7)
Febrile neutropenia	40 (8.9)	59 (6.0)	99 (6.9)
Leukopenia	23 (5.1)	68 (6.9)	91 (6.4)
Anaemia	27 (6.0)	43 (4.4)	70 (4.9)
Fatigue	18 (4.0)	44 (4.5)	62 (4.3)
Diarrhoea	14 (3.1)	38 (3.9)	52 (3.6)

TEAE, treatment-emergent adverse event.

Dose delay occurred in 619 patients (43.2%), while dose reduction (from 25 mg/m^2^ to 20 mg/m^2^) occurred in 316 patients (22.1%); toxicity related to cabazitaxel was the cause of dose delay in 279 patients (19.5%) and dose reduction in 277 patients (19.3%). The most frequently occurring TEAEs leading to premature discontinuation of cabazitaxel were fatigue (2.1%), febrile neutropenia (1.5%), anaemia (1.1%), diarrhoea (0.9%), acute renal failure (0.8%), urinary tract infection (0.8%) and neutropenia (0.8%). The most frequently occurring TEAEs leading to death (regardless of causality), were disease progression (1.3%), acute renal failure (0.3%), renal failure (0.3%), general physical health deterioration (0.3%), sepsis (0.3%), pneumonia (0.3%) and febrile neutropenia (0.3%). Forty-one patients (2.9%) died due to a TEAE possibly related to treatment; the most frequently occurring treatment-related TEAEs leading to death were febrile neutropenia and pneumonia (4 patients, 0.3% each).

## DISCUSSION

The CUP/EAP studies succeeded in their primary objective of providing patients with progressive mCRPC access to cabazitaxel from 2010 onwards in countries where it was not yet commercially available. In doing so, these studies fulfilled an important unmet medical need for patients progressing during or after docetaxel. In CUP/EAP, 33.8% of patients had progressed within 3 months of last docetaxel dose (16.6% had progressed during docetaxel therapy itself). Around 1 in 4 patients (24.2%) received 10 or more cycles of cabazitaxel, indicating a long duration of treatment in some patients and suggesting clinical benefit, despite a current lack of overall survival data.

Cabazitaxel had a manageable safety profile in this study. Of note, this study included elderly patients (≥ 75 years of age) and patients with ECOG PS 2 (21.2% and 8.9% of population, respectively). Moreover, the most frequently reported TEAEs were consistent with the safety profile of cabazitaxel reported in the TROPIC and PROSELICA Phase III studies [7, 10]. Of note, the rates of clinical neutropenia and febrile neutropenia were similar between CUP/EAP and TROPIC. The previously published incidences of Grade 3/4 neutropenia were based on laboratory assessments (82% in TROPIC and 73% in PROSELICA) [7, 10]. The rates of Grade 3/4 neutropenia as a symptomatic, clinical AE were 18.7% in CUP/EAP vs. 21.3% in TROPIC (Grade 3/4 febrile neutropenia: 6.9% vs. 7.5%, respectively) (data on file) [7]. In addition, only 5 patients (0.3%) had Grade 3/4 peripheral neuropathy, consistent with the low incidence of peripheral neuropathy seen in previous studies.

In TROPIC, prophylactic use of G-CSF was not permitted during the first cycle, but was allowed after the first occurrence of either neutropenia lasting ≥ 7 days, or neutropenia complicated by fever or infection [7]. In CUP/EAP, 64.0% of patients received G-CSF at any cycle, and 53.9% of patients received G-CSF at Cycle 1. Prophylactic use of G-CSF may have lowered neutropenia rates in CUP/EAP, consistent with previous findings [13]. Furthermore, the risk of neutropenia with cabazitaxel treatment should be re-evaluated in the light of recent data suggesting that the 20 mg/m^2^ cabazitaxel dose is non-inferior, in terms of overall survival, to the 25 mg/m^2^ dose (PROSELICA) [10].

Despite the observed manageable safety profile in CUP and EAP, it is important to note that these studies are associated with certain limitations. Because the CUP/EAP studies were conducted across 41 countries, the impact of regional variation should be considered. In some countries, for example, longer treatment duration (i.e. a higher number of cycles) was considered routine practice and undertaken more often than in other countries. In addition, variation in regional guidelines for the prophylactic and therapeutic use of G-CSF for neutropenia may have influenced safety outcomes, despite the recommendations issued to the investigators. A further limitation of this pooled analysis is that efficacy data from the EAP study were not taken into account.

This global, real-world pooled analysis of CUP/EAP further demonstrates the manageable safety profile of cabazitaxel and supports its use as a treatment option for patients with mCRPC, including those who are refractory or unresponsive to first-line treatment with docetaxel [15]. A variety of other strategies and novel agents are available, but new potential mechanisms of resistance to these agents are emerging. Therefore, cabazitaxel remains an important treatment option for patients with mCRPC.

## MATERIALS AND METHODS

### Study design and treatment protocol

CUP and EAP were international, multicentre, prospective, open-label registry studies. Patients were enrolled across 41 countries and treated with cabazitaxel until the occurrence of disease progression, death, unacceptable toxicity, a decision by the physician or patient refusal of further treatment (in some countries, the availability of new treatments may also have led to the discontinuation of cabazitaxel). In Germany, patients were no longer able to continue with the study once cabazitaxel was made commercially available, as per local regulations; patients who continued with treatment subsequently received the marketed drug.

On Day 1 of each cycle, patients received cabazitaxel at a dose of 25 mg/m², administered intravenously over a 1-hour period. In addition, patients received oral prednisone or prednisolone 10 mg daily throughout each cycle, as well as a corticosteroid (dexamethasone 8 mg or equivalent) at least 30 minutes prior to each administration of cabazitaxel. G-CSF was administered as primary (Cycle 1) and secondary prophylaxis (later cycles), as per ASCO guidelines (to prevent neutropenic events), or was administered therapeutically (to treat neutropenic events). Physicians were advised to consider primary prophylaxis with G-CSF in patients with high-risk clinical features (age ≥ 65 years, poor performance status, previous episodes of febrile neutropenia, extensive prior radiation ports, poor nutritional status or other serious comorbidities) that typically predispose patients to increased complications from prolonged neutropenia. The length of each cabazitaxel cycle was 3 weeks (± 3 days). New cycles were delayed until an absolute neutrophil count ≥ 1500/mm^3^, platelet count ≥ 75,000/mm^3^ and non-haematological toxicities (except alopecia) had recovered to baseline. A maximum of 2 weeks’ delay was permitted between any 2 treatment cycles. The cabazitaxel dose could be reduced to 20 mg/m^2^ in cases of toxicity; however, once reduced, the dose was not to be re-escalated.

The primary objective was to allow access to cabazitaxel before its commercial availability in patients with mCRPC whose disease had progressed during or after docetaxel treatment, and who had similar disease and baseline characteristics to patients in the TROPIC trial. The secondary objective was to assess the overall safety of cabazitaxel in these patients. In some countries, HRQoL data were also collected (not reported here). The studies were conducted in accordance with the principles outlined in the Declaration of Helsinki (18th World Medical Assembly, 1964) and all its subsequent amendments. Each patient provided signed, written, informed consent before enrolment.

### Patient population

Patients eligible for enrolment were ≥ 18 years of age, and had a life expectancy > 3 months, an ECOG PS of ≤ 2, mCRPC that had progressed during or after treatment with a docetaxel-containing regimen, adequate bone marrow, liver and renal function, and prior surgical or medical castration. Standard demographic and baseline characteristics (including age, height and weight), medical and surgical history, metastatic sites, cancer diagnosis and prior docetaxel therapy were collected at baseline.

Ineligibility criteria included a history of severe hypersensitivity reaction (Grade ≥ 3) to docetaxel or Polysorbate 80-containing drugs, intolerance or hypersensitivity to prednisone/prednisolone, active Grade ≥ 2 peripheral neuropathy or stomatitis, prior radiotherapy to ≥ 40% of bone marrow, or prior therapy with certain radionuclides.

### Safety assessments

Safety assessments included analysis of TEAEs and SAEs. The study protocol defined a TEAE as any untoward medical event occurring (or worsening) during the on-treatment period (from the first day of cabazitaxel administration up to 30 days after the last administration), which did not necessarily have a causal relationship with the treatment; an SAE was defined as any untoward medical occurrence that resulted in death, was life threatening, required hospitalisation, or resulted in persistent or significant disability/incapacity. Laboratory, vital sign or electrocardiogram abnormalities were recorded as TEAEs only if medically relevant (symptomatic, requiring corrective treatment, leading to discontinuation and/or classified as serious). AEs were graded according to the National Cancer Institute Common Terminology Criteria for Adverse Events, version 4.0. Monitoring of complete blood counts was performed on a weekly basis during Cycle 1 and before each treatment cycle thereafter to allow for dose adjustment, if needed.

## Abbreviations

ASCO: American Society of Clinical Oncology
CUP: compassionate use programme
EAP: early access programme
ECOG PS: Eastern Cooperative Oncology Group performance status
G-CSF: granulocyte colony-stimulating factor
HRQoL: health-related quality of life
mCRPC: metastatic castration-resistant prostate cancer
SAE: serious adverse event
TEAE: treatment-emergent adverse event

## References

[1] Ferlay J, Soerjomataram I, Dikshit R, Eser S, Mathers C, Rebelo M, Parkin DM, Forman D, Bray F. Cancer incidence and mortality worldwide: sources, methods and major patterns in GLOBOCAN 2012. Int J Cancer. 2015; 136:E359–86. 10.1002/ijc.29210. 25220842

[2] Kirby M, Hirst C, Crawford ED. Characterising the castration-resistant prostate cancer population: a systematic review. Int J Clin Pract. 2011; 65:1180–1192. 10.1111/j.1742-1241.2011.02799.x. 21995694

[3] National Cancer Institute. Cancer Stat Facts: Prostate Cancer. 2018 Available from: https://seer.cancer.gov/statfacts/html/prost.html.

[4] National Prostate Cancer Adult (NPCA). Second Year Annual Report – Further analysis of existing clinical data and preliminary results from the NPCA Prospective Audit. 2015 Available from: https://www.npca.org.uk/content/uploads/2018/02/NPCA-2015-Annual-Report_FINAL_301115.pdf.

[5] Tannock IF, de Wit R, Berry WR, Horti J, Pluzanska A, Chi KN, Oudard S, Theodore C, James ND, Turesson I, Rosenthal MA, Eisenberger MA. Docetaxel plus prednisone or mitoxantrone plus prednisone for advanced prostate cancer. N Engl J Med. 2004; 351:1502–1512. 10.1056/NEJMoa040720. 15470213

[6] Petrylak DP, Tangen CM, Hussain MH, Lara PN Jr, Jones JA, Taplin ME, Burch PA, Berry D, Moinpour C, Kohli M, Benson MC, Small EJ, Raghavan D, et al. Docetaxel and estramustine compared with mitoxantrone and prednisone for advanced refractory prostate cancer. N Engl J Med. 2004; 351:1513–1520. 10.1056/NEJMoa041318. 15470214

[7] de Bono JS, Oudard S, Ozguroglu M, Hansen S, Machiels JP, Kocak I, Gravis G, Bodrogi I, Mackenzie MJ, Shen L, Roessner M, Gupta S, Sartor AO. Prednisone plus cabazitaxel or mitoxantrone for metastatic castration-resistant prostate cancer progressing after docetaxel treatment: a randomised open-label trial. Lancet. 2010; 376:1147–1154. 10.1016/S0140-6736(10)61389-X. 20888992

[8] Bahl A, Oudard S, Tombal B, Ozguroglu M, Hansen S, Kocak I, Gravis G, Devin J, Shen L, de Bono JS, Sartor AO. Impact of cabazitaxel on 2-year survival and palliation of tumour-related pain in men with metastatic castration-resistant prostate cancer treated in the TROPIC trial. Ann Oncol. 2013; 24:2402–2408. 10.1093/annonc/mdt194. 23723295 PMC3755329

[9] Heidenreich A, Scholz HJ, Rogenhofer S, Arsov C, Retz M, Muller SC, Albers P, Gschwend J, Wirth M, Steiner U, Miller K, Heinrich E, Trojan L, et al. Cabazitaxel plus prednisone for metastatic castration-resistant prostate cancer progressing after docetaxel: results from the German compassionate-use programme. Eur Oncol. 2013; 63:977–982. 10.1016/j.eururo.2012.08.058. 23116658

[10] Eisenberger M, Hardy-Bessard AC, Kim CS, Geczi L, Ford D, Mourey L, Carles J, Parente P, Font A, Kacso G, Chadjaa M, Zhang W, Bernard J, et al. Phase III Study Comparing a Reduced Dose of Cabazitaxel (20 mg/m^2^) and the Currently Approved Dose (25 mg/m^2^) in Postdocetaxel Patients With Metastatic Castration-Resistant Prostate Cancer-PROSELICA. J Clin Oncol. 2017; 35:3198–3206. 10.1200/JCO.2016.72.1076. 28809610

[11] Sanofi. JEVTANA^©^ (cabazitaxel) Injection, Summary of Product Characteristics, EMA. 2017 Available from: https://www.medicines.org.uk/emc/product/4541/smpc.

[12] Bracarda S, Gernone A, Gasparro D, Marchetti P, Ronzoni M, Bortolus R, Fratino L, Basso U, Mazzanti R, Messina C, Tucci M, Boccardo F, Carteni G, et al. Real-world cabazitaxel safety: the Italian early-access program in metastatic castration-resistant prostate cancer. Future Oncol. 2014; 10:975–983. 10.2217/fon.13.256. 24295376

[13] Heidenreich A, Bracarda S, Mason M, Ozen H, Sengelov L, Van Oort I, Papandreou C, Fossa S, Hitier S, Climent MA, and European investigators. Safety of cabazitaxel in senior adults with metastatic castration-resistant prostate cancer: results of the European compassionate-use programme. Eur J Cancer. 2014; 50:1090–1099. 10.1016/j.ejca.2014.01.006. 24485664

[14] Di Lorenzo G, D’Aniello C, Buonerba C, Federico P, Rescigno P, Puglia L, Ferro M, Bosso D, Cavaliere C, Palmieri G, Sonpavde G, De Placido S. Peg-filgrastim and cabazitaxel in prostate cancer patients. Anticancer Drugs. 2013; 24:84–89. 10.1097/CAD.0b013e32835a56bc. 23044721

[15] Di Lorenzo G, Bracarda S, Buonerba C, Aieta M, Mirone V. Poor survival in prostate cancer patients with primary refractoriness to docetaxel. Eur Urol. 2014; 65:505–507. 10.1016/j.eururo.2013.10.037. 24211139

